# The Editor's Letter-Box

**Published:** 1916-06-10

**Authors:** 


### Economy in the Annual Report.

SIR,—In matters of charity finance one must be a purist or run the risk of being classed with the suspect. No purist can agree to anything but full publication of accounts. Such publication includes the free sending of an unsolicited copy to every person entitled to receive it. It is not sufficient to issue a statement to the effect that a copy will be sent if required. This would be equal to saying that certain action, which requires the concurrence of the notified person, will be carried out unless objection is made. Accounts in the hospital sense include the lists of donations or subscriptions, the totals of which are shown in the income and expenditure account. No motive of economy can justify the withholding of free circulation, and no professional hospital secretary giving a considered opinion would agree to such withholding.

Imagine therefore, Sir, the feelings of one who for many years has regarded you as the chief exponent of pure financial principles as applied to hospital accounts when reading the note on this subject under "Hospital and Institutional News" in THE HOSPITAL of June 3, where those who are responsible for the cheeseparing at St. Mary's and Charing Cross are praised for what appears to many hospital men an indefensible action.

If economy is what is aimed at by all means give only essentials in the report if that is considered advisable, and print the matter supplied on as thin paper as possible in order to save postage. This course might even be beneficial as resulting in the scrapping of features which have ceased to be valuable or interesting. But not to circulate essential information is ethically wrong and stupidly impolitic.

The report is a money-raising instrument, and people like to see their contributions recorded in the orthodox manner. The amount of money gained by this instance of false economy will be lost several times over in renewals withheld by those who have missed the customary stimulation to their interest and necessary recognition of their generosity.—I am, Sir, your obedient servant,

A LONDON SECRETARY.

[Our readers should realise fully the importance of the qualification of all attempts at economy in the annual report set forth in the note on page 164 of THE HOSPITAL of May 20, 1916. It is not economy to set up the whole report in full at great cost and then to strike off only a few copies, giving the governors a mere intimation that they can obtain one on application to the secretary, instead of forwarding them a copy as heretofore.—ED. THE HOSPITAL.]

